# Investigation of MnSc_2_X_4_ (X = S, Se) spinels to unveil their potential for optoelectronic and thermoelectric applications

**DOI:** 10.1039/d4ra08334b

**Published:** 2025-03-31

**Authors:** Syed Ali Raza, Ghulam M. Mustafa, M. Adil Ameer, N. A. Noor, Zahid Farooq, Sohail Mumtaz, Ihab Mohamed Moussa

**Affiliations:** a Department of Physics, Riphah International University Lahore Campus Pakistan; b Department of Physics, Division of Science and Technology, University of Education Lahore Punjab 54770 Pakistan dr.ghulam.muhammad@ue.edu.pk; c Department of Physics, University of Sargodha 40100 Sargodha Pakistan; d Department of Chemical and Biological Engineering, Gachon University 1342 Seongnamdaero Sujeong-gu Seongnam-si 13120 Republic of Korea sohail38@gachon.ac.kr; e Department of Botany and Microbiology, College of Science, King Saud University P. O. Box 2455 Riyadh 11451 Saudi Arabia

## Abstract

To advance the fields of optoelectronics and thermoelectrics, the demand for developing novel materials with exceptional optoelectronic and thermoelectric characteristics has greatly increased. In this respect, spinels are among the well-researched materials in the scientific community. The present study examines the mechanical, optoelectronic, magnetic, and thermoelectric characteristics of MnSc_2_X_4_ (X = S, Se), utilizing the WIEN2k and BoltzTrap code. The investigated materials exhibit cubic structures with the *Fd*3̄*m* space group and lattice parameters of 10.44 and 10.94 Å for S- and Se-based compositions, respectively. Fulfillment of the Born stability criteria attest to the mechanical stability, while negative formation energies validate the thermodynamic stability of the spinels. The spin-polarized electronic band structure (EBS) and the density of states (DOS) reveal the semiconducting nature of the materials with direct bandgap (*E*_g_). In the optical parameters, MnSc_2_S_4_ and MnSc_2_Se_4_ show a static dielectric constant of 6.5 and 8.5, respectively. The materials exhibit wide absorption spectra, ranging from visible to ultraviolet (UV) regions, with the highest absorption in the UV region. The spin-dependent volume optimization demonstrates the ferromagnetic (FM) nature of the materials. The presence of Mn ions in the compositions imparts this magnetic nature to them, which is analyzed by its high local magnetic moment. Lastly, the transport characteristics are analyzed against the increasing temperature in the range of 300–800 K. These materials exhibit high Seebeck coefficients of 242 to 251 μV K^−1^, and their electrical and thermal conductivities increase with temperature. The figure of merit *ZT* showed an almost constant value at varying temperatures. The outcomes of this study demonstrate the investigated materials as potential candidates for optoelectronic and thermoelectric applications.

## Abstract 2

Structural, optical, and mechanical insight of MnSc_2_X_4_ (X = S, Se) spinels.

## Graphical abstract

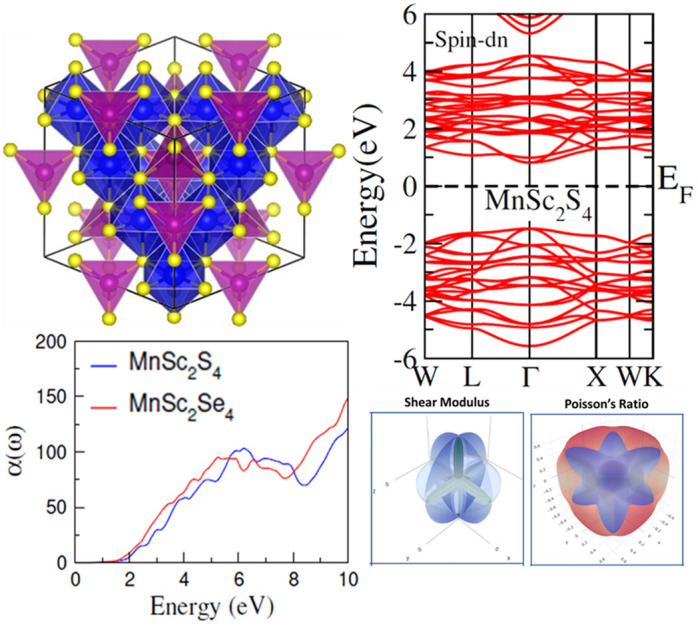

## 1. Introduction

Optoelectronics is a rapidly growing field that relies on the quantum mechanical interaction between light and electronic materials.^1^ It is now extensively applied in the fields of energy harvesting,^1^ light and display technology,^2^ sensors,^3^ medical,^4^ and communication technology.^5^ Recent advances in the field of optoelectronics include the consequences of the discovery of such new materials that exhibit structural and thermal stability, tunable electronic properties, and high quantum and power conversion efficiencies. In this regard, spinels are emerging materials that not only fulfill the stability criteria^6,7^ but also provide promising optoelectronic characteristics.^8,9^ The spinel compounds have a general compositional formula of AB_2_X_4_ and possess a crystal structure similar to that of the MgAl_2_O_4_ mineral. In their formula unit, the A and B sites are occupied by cations tetrahedrally and octahedrally coordinated with the X anion, respectively. The A-site cation is typically an alkali or alkaline earth metal or transition metal, and the B-site cation is usually a transition metal. Meanwhile, the X-site comprises chalcogenides or halogen.^10^

Spinel compounds generally cover a wide range of electronic bandgaps (*E*_g_) and show diverse optical properties. Furthermore, the spinels provide the flexibility of modifying their bandgap and other electronic and optical properties by means of substitution or doping.^11,12^ Owing to these properties, spinels have been explored as a potential candidate for various applications like solar cells,^13^ LEDs,^14^ electroluminescence, electro-photocatalysis,^15^ and laser diodes.^16^ In addition to optoelectronic characteristics, spinels are known for their exceptional thermoelectric and transport properties. Many spinel compounds exhibit high electrical and low thermal conductivity with a high Seebeck coefficient, making them desirable for thermoelectric applications.^17^

Among the spinels, the most investigated compositions comprise chalcogenide-based spinel compounds. The chalcogenide-based compositions exhibit mechanical and thermodynamic stability, which are essential parameters for device fabrication. Regarding the stability of chalcogenide spinels, Noor *et al.* investigated the spinels HgSm_2_X (X = S and Se) and reported the negative enthalpy of formation for these materials, which verified their thermodynamic stability. The reported values of elastic constants fulfilled the mechanical stability criteria.^18^ In a related investigation, Mustafa *et al.* revealed mechanical and thermodynamic stability in the HgY_2_X_4_ (X = S, Se). Furthermore, the materials were found to exhibit a direct electronic bandgap with a magnitude of 1.2 and 0.6 eV for S and Se-based compositions, respectively.^19^ In another study on the tuning of the bandgap, Shahid *et al.* experimentally prepared the samples of Mg_1−*x*_Sr_*x*_Al_2_O_4_ (*x* = 0.1 to 1.0) and Mg_1−*x*−*y*_Sr_*x*_Mn_*y*_Al_2_O_4_ (*x* = 0.1 to 0.7 and *y* = 0.1), and reported on the effect of Sr^2+^ and Sr^2+^/Mn^2+^ doping at the A-site. It was found that for Sr^2+^ doping, the compositions revealed bandgap values in the range of 4.7 to 5 eV. However, the co-doping of Mn^2+^ resulted in a decrease in the bandgap, and gave the values in the range of 2.54 to 3.14 eV.^20^ In a comprehensive study of the optoelectronic properties of spinels, Tahir *et al.* investigated MgSc_2_(S/Se)_4_ and reported direct bandgaps of 2.3 eV and 1.7 eV for S-based and Se-based compositions, respectively. The materials revealed high absorption in the visible and UV regions of the spectrum. In the UV regions, the materials have an absorption coefficient on the order of 10^6^ cm^−1^. The materials were also investigated for their thermoelectric characteristics, and the results demonstrated that materials exhibited high Seebeck coefficient in the range of 220 to 235 μV K^−1^.^21^ In a separate study on the thermoelectric properties of chalcogenide spinels, Sameeullah *et al.* explored the spinel compounds Ln_2_MnSe_4_ (Ln = Yb, Lu). They reported that on substituting Yb with Lu in the composition, its thermoelectric performance was improved and the figure of merit values increased from 0.18 to 0.79.^22^

Inspired by the above-mentioned studies on the optoelectronic and thermoelectric characteristics of spinels, we computationally simulated the chalcogenide-based spinels MnSc_2_X_4_ (X = S and Se) on DFT-based WIEN2k software and investigated their mechanical, electronic and optical characteristics. Furthermore, the current work also includes the transport and thermoelectric investigations of the materials which were carried out by employing BoltzTrap code. The results of the present study are promising, and are a worthwhile addition to our current understanding of the properties of spinels, demonstrating the potential of the examined materials for the applications of optoelectronics and thermoelectrics.

## 2. Computational details

In the present investigations, DFT-based WIEN2k software was utilized to explore the mechanical, opto-electronic and magnetic characteristics of MnSc_2_X_4_ (X = S, Se). Self-consistency calculations were executed using the full-potential linearized augmented plane wave (FP-LAPW) approach. PBE-GGA was used to estimate the exchange–correlation potential. However, it is not very efficient in accurately computing the electronic properties. To eliminate its error, the EBS and DOS were calculated by using the mBJ potential because it provides relatively greater accuracy in the measurement of electronic properties.^23^ Moreover, the EBS was further computed using HSE06. To determine the most favorable and lowest ground state of the spinel MnSc_2_X_4_, the ferromagnetic (FM) and anti-ferromagnetic (AFM) configurations for MnSc_2_S_4_ and MnSc_2_Se_4_ were examined in detail. In this analysis, the unit cell that we studied in WIEN2k comprises four S/Se atoms in the MnSc_2_X_4_ structure. The ferromagnetic case was studied considering all the atoms with up-spin orientation, resulting in a net magnetic moment. It is worth noting that a high contribution in magnetic moment arises from the Mn atoms, and the other atoms showed almost a negligible contribution in the total magnetic moment of the unit cell. Keeping this in mind, anti-ferromagnetic orientations were only applied to the four Se atoms assigning the two atoms with up-spin orientation and two with down-spin configuration, resulting in a zero net magnetic moment of the unit cell. Zero net magnetization confirms the true anti-ferromagnetic orientations of the Se-atoms. This orientation of spins in the magnetic lattice allowed for an observable contrast in the electronic and magnetic properties of the spinel. By comparing the two energies, we found the ferromagnetic configuration to be in a more negative range, confirming the ferromagnetic stability of our compound.

The FP-LAPW approach treats the unit cell as two distinct sections: the muffin-tin (MT) or non-overlapping atomic spheres and the interstitial region. The solution of the wavefunction in the MT region was assumed to be spherically harmonic, whereas for the interstitial region, a plane-wave solution was considered. Some parameters remained constant throughout the computation, which includes the size of the basic set (*R*_MT_ × *K*_max_), where *R*_MT_ symbolizes the radius of atomic spheres, and *K*_max_ depicts the plane wave cutoff. The magnitude of *R*_MT_ × *K*_max_ is set at 8. The other parameter is the angular momentum 
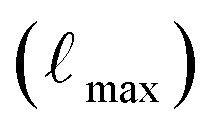
, which is chosen as 10. The Fourier expansion of charge densities *G*_max_ was selected as 20. For the sake of good convergence, a mesh of 12 × 12 × 12 was selected in the first Brillion zone, and 10^−2^ mRy was chosen as the energy cutoff. Lastly, the transport parameters were computed using semi-classical Boltzmann theory with constant relaxation time (*τ* = 10^−14^ s), and the BoltzTrap and ShengBTE code were utilized to execute the transport and thermoelectric calculations.

## 3. Results and discussions

### 3.1 Structural properties

**Fig. 1:**
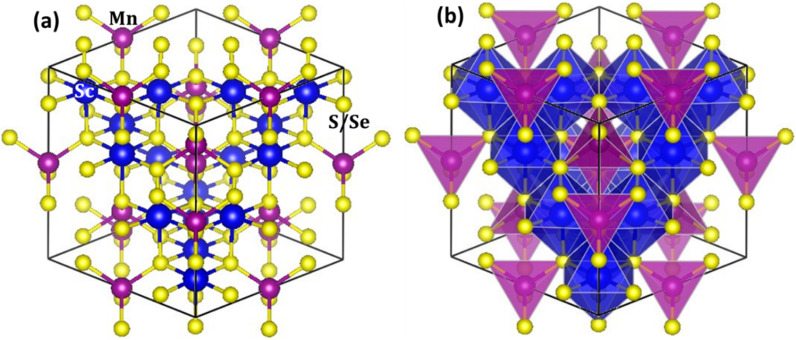
Unit cell structure of MnSc_2_X_4_ (X = S, Se) (a) ball-stick format and (b) polyhedral model. The Mn, Sc and S/Se ions are displayed with purple, blue and yellow spheres, respectively.

The unit cell structure of the MnSc_2_X_4_ (X = S, Se) spinels are presented in Fig. 1. Fig. 1(a) and (b) represent the molecular model, including the ball-stick and polyhedral representation of the investigated materials, in which the Mn, Sc, and S/Se ions are portrayed by purple, blue and yellow balls, respectively. Furthermore, in Fig. 1, the FCC cubic structure of the unit cells with *Fd*3̄*m* space group can be seen, in which Mn has a tetrahedral coordination with S/Se, whereas Sc has an octahedral coordination with S/Se. In the unit cell, the Mn, Sc and S/Se ions have the oxidation states of +2, +3, and −2 and lie at the lattice positions of (0.125, 0.125, 0.125), (0.5, 0, 0) and (0.25, 0.25, 0.25), respectively.^24^

**Fig. 2:**
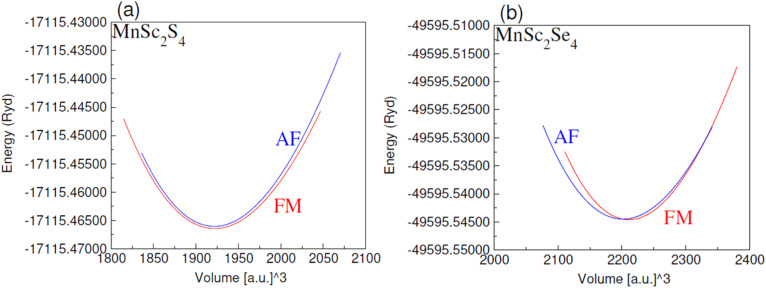
Computed plots of energy (Ryd) relative to volume ([a.u.]^3^) for spinels (a) MnSc_2_S_4_, (b) MnSc_2_Se_4_ in AFM and FM spin-orientations.

To compute the lattice constants (*a*_0_), the unit cell volume was optimized for the lowest ground state energy. The data obtained from the volume optimization were then fitted in the Birch Murnaghan equation to obtain the smooth curves, which are presented in Fig. 2. From the optimized unit cell volume, we computed the *a*_0_ and listed their magnitudes in Table 1, which are 10.44 Å and 10.94 Å for MnSc_2_S_4_ and MnSc_2_Se_4_, respectively. It can be seen that the lattice constants increase when S in the unit cell is replaced by Se. This is because Se has a comparatively larger ionic radius than S, so when substituted, the unit cell volume rises and the lattice constants subsequently increase. Furthermore, the volume optimization was utilized to identify the magnetic nature of the spinels. For this purpose, the unit cell volume was optimized sequentially with ferromagnetic (FM) and antiferromagnetic (AFM) spin phases. It can be seen from Fig. 2 that the unit cell with FM spin phase shows lower energy, which shows that the materials are more stable in their FM phase. To probe the thermodynamic stability of the investigated spinels, the formation energies (Δ*H*_f_) of each composition was calculated. The Δ*H*_f_ represents the difference of energy between the composition and its constituent ions. In the current instance, it can be written as:1
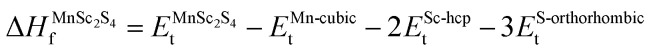
2
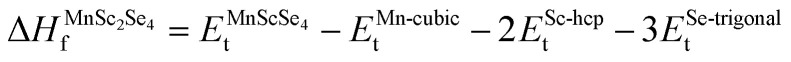
In the preceding relation, the terms 
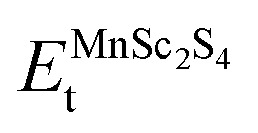
 and 
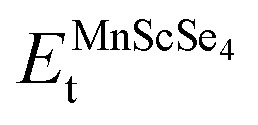
 depict the total energy of the compound, while *E*^Mn-cubic^_t_, *E*^Sc-hcp^_t_, *E*^S-orthorhombic^_t_ and *E*^Se-trigonal^_t_ represent the most stable energy values of the constituent elements of Mn, Sc, S and Se, respectively. Both spinel compositions revealed a negative magnitude of Δ*H*_f_, which shows that their formation involves the exothermic reaction and heat evolves in the formation process. The evolution of heat makes the compounds have less energy than their constituents, which demonstrates their thermodynamic stability. Table 1 tabulates the magnitude of Δ*H*_f_, which is −2.86 and −1.93 eV for MnSc_2_S_4_ and MnSc_2_Se_4_, respectively. From the magnitude of Δ*H*_f_ of both compositions, it can be seen that the formation of MnSc_2_S_4_ involves a greater evolution of energy, which means that it is comparatively more thermodynamically stable.

**Table 1:** Optimized magnitudes of *a*_0_ (Å), *B*_0_ (GPa), Δ*H*_f_ (eV), and elastic parameters, calculated for MnSc_2_S/Se_4_

Composition	*a*_0_ (Å)	*B*_0_ (GPa)	Δ*H*_f_ (eV)	*C*_11_	*C*_12_	*C*_44_	*B*_0_	*G*	*Y*	*ν*	*B*_0_/*G*
MnSc_2_S_4_	10.44	86.35	−2.86	142.71	51.26	23.16	81.74	30.52	81.42	0.37	2.68
MnSc_2_Se_4_	10.94	71.45	−1.93	135.63	30.76	10.04	65.72	20.92	56.74	0.32	3.14

### 3.2 Mechanical properties

It is highly critical to analyze the mechanical characteristics of the compounds because they directly influence the long-term performance of the fabricated device. To evaluate the mechanical characteristics of MnSc_2_S_4_ and MnSc_2_Se_4_, we computed their elastic stiffness constants, which provide information on the material's behavior to the applied stress. Since the examined spinels exhibit cubic symmetry, the elastic stiffness constants required for the computation are reduced to three: *C*_11_, *C*_12_, and *C*_44_. Furthermore, the mechanical stability of these cubic crystals can be examined by means of the widely known Born stability criteria. As per the criteria, these cubic spinels are considered mechanically stable if they meet the following requirements:^25^

3 *C*_44_ > 0, *C*_11_ − *C*_12_ > 0, *C*_11_ + 2*C*_12_ > 0

Table 1 tabulates the calculated values of *C*_11_, *C*_12_, and *C*_44_, which satisfy the above-stated necessary conditions for mechanical stability. These elastic stiffness constants (*C*_*ij*_) are very useful in computing the elastic moduli, which incorporate the shear modulus (*G*), bulk modulus (*B*_0_), and Young's modulus (*Y*). Through these *C*_*ij*_, we computed the elastic moduli by means of the following relations:^26^

4 *B*_0_ = (*C*_11_ + 2*C*_12_)/3

The magnitude of *G* is the averaged value of Voigt's and Reuss's shear modulus denoted by *G*_V_ and *G*_R_, respectively, and their relations are written below:

5 *G*_v_ = (3*C*_44_ + *C*_11_ − *C*_12_)/5 *G*_R_ = [5*C*_44_(*C*_11_ − *C*_12_)]/[4*C*_44_ + 3(*C*_11_ − *C*_12_)]

6 *G* = (*G*_R_ + *G*_V_)/2

Once the magnitudes of *G* and *B*_0_ have been measured, then the value of *Y* can be computed by using the equation given below:

7 *Y* = (9*B*_0_*G*)/(3*B*_0_ + *G*)

Aside from computing *B*_0_ with *C*_*ij*_, the present study also reports the magnitude of *B*_0_ calculated by inserting the data of the optimized volume in the Birch–Murnaghan equation. From the computed values of the elastic moduli, it is apparent that substituting Se in the composition causes a decrease in the elastic moduli, which means that MnSc_2_Se_4_ is less rigid relative to MnSc_2_S_4_. This is due to the relatively lower difference in the electronegativity between Se and the constituent cations. The lower electronegativity difference causes a decrease in the bond strength, which in turn is accountable for the decreases in the elastic moduli.^27^ The Pugh's and Poisson's ratios denoted by *B*_0_/*G* and *ν* for both materials, respectively, were also calculated. These values provide information on their ductile or brittle nature. A material with *B*_0_/*G* > 1.75 and *ν* > 0.26 is recognized as ductile; if not, then it is considered as brittle material.^28,29^ From the computed magnitude of these ratios, the investigated compositions are considered as ductile materials. Another approach that can be utilized to attest to the ductile nature is Cauchy's pressure, which is computed by *C*_12_ − *C*_44_. The ductile materials give a positive magnitude of Cauchy's pressure, whereas the negative Cauchy pressure represents the brittle nature.^30^ In the case of the investigated compositions, the positive Cauchy's pressure further validates the ductile character of the two spinels. The computed magnitudes of the elastic moduli and Pugh's and Poisson's ratios are summarized in Table 1.

**Fig. 3:**
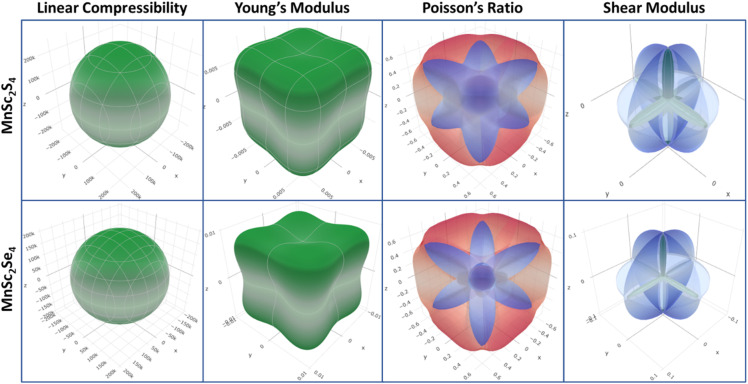
3-D illustration of the linear compressibility (*β*), elastic moduli (*Y* and *G*) and Poisson's ratio (*v*) for MnSc_2_X_4_ (X = S, Se).

Many crystal materials are not elastically isotropic and have different magnitudes of elastic parameters in different dimensions. The investigated materials show similar behavior and are anisotropic in nature. Fig. 3 depicts the 3-D illustration of the magnitudes of linear compressibility (*β*), shear modulus (*G*), Young's modulus (*Y*), and Poisson's ratio (*ν*). It is apparent that except for *β*, the plots of all the remaining parameters have a non-spherical shape, which reveals that their magnitude varies spatially. Furthermore, we computed the numerical value of anisotropy (*A*) using the ratio of the maximum-to-minimum magnitudes of the parameters. In the case of *A* = 1, the composition is recognized as isotropic. Meanwhile, in another case, the material is considered anisotropic. The computed values of *A* reveal the isotropic behavior only for linear compressibility, as seen in Fig. 3. It is noted that the magnitude of *A* for the Young's and shear moduli increases when Se is substituted in place of S in the composition. However, the anisotropy of Poisson's ratio is relatively greater in MnSc_2_S_4_.

### 3.3 Electronic properties

**Fig. 4:**
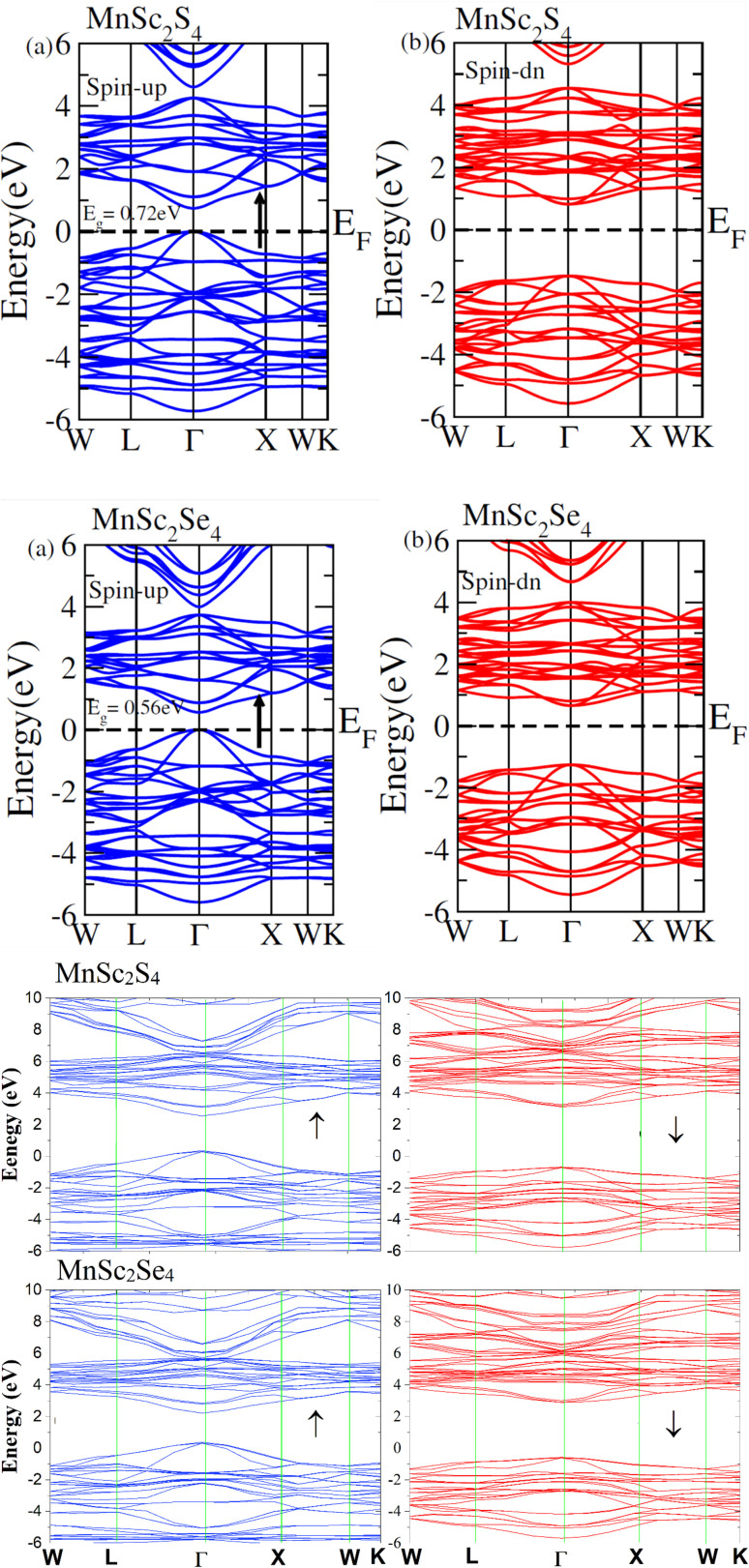
(a) Electronic band structures for spinels MnSc_2_X_4_ (X = S, Se), computed with the mBJ potential. (b) Electronic band structures for spinels MnSc_2_X_4_ (X = S, Se), computed with HSE06.

Investigations of the electronic characteristics involve an understanding of the electronic conduction mechanism. To this end, we analyzed the electronic conduction mechanism of the investigated materials through their spin-polarized EBS and DOS. The DOS were calculated for energies ranging from −6 eV to +6 eV, whereas the EBS was computed from −6 eV to +6 eV and −6 eV to +10 eV relative to the Fermi level (*E*_F_). Fig. 4(a) and (b) shows the spin-polarized EBS of MnSc_2_S_4_ and MnSc_2_Se_4_, and the plots in the figure depict different band structures in different spin states computed *via* mBJ potential and HSE-06. The EBS revealed that for both spin channels, *E*_F_ occurs between the valence band (VB) and conduction band (CB). This demonstrates the semiconducting nature of the investigated spinels. Moreover, both compositions have a direct bandgap (*E*_g_) because, in both spin configurations, the maxima of VB and minima of CB lie at the same high-symmetry *Γ* point. In the up-spin configuration, MnSc_2_S_4_ and MnSc_2_Se_4_ showed a narrow bandgap with the magnitudes of 0.72 and 0.56 eV, respectively. Also, when we employed HSE-06, both compositions revealed semiconducting character with a direct band gap. The calculated bandgaps are 2.2 eV and 1.9 eV for MnSc_2_S_4_ and MnSc_2_Se_4_, respectively. Conversely, in the down-spin channel, the electronic states of the VB maxima are well below the *E*_F_, which results in an increase in the bandgap. Fig. 4 clearly shows that MnSc_2_Se_4_ has a smaller *E*_g_ as compared to MnSc_2_S_4_. The additional electronic states in Se and its relatively lower electronegativity difference with cations account for this smaller *E*_g_.^31,32^ The additional electronic states increase the band size, and the lower electronegativity difference results in its electronic states being less localized. This causes the shifting of electronic bands toward *E*_F_, and consequently leads to the reduction in the bandgap.^33^

**Fig. 5:**
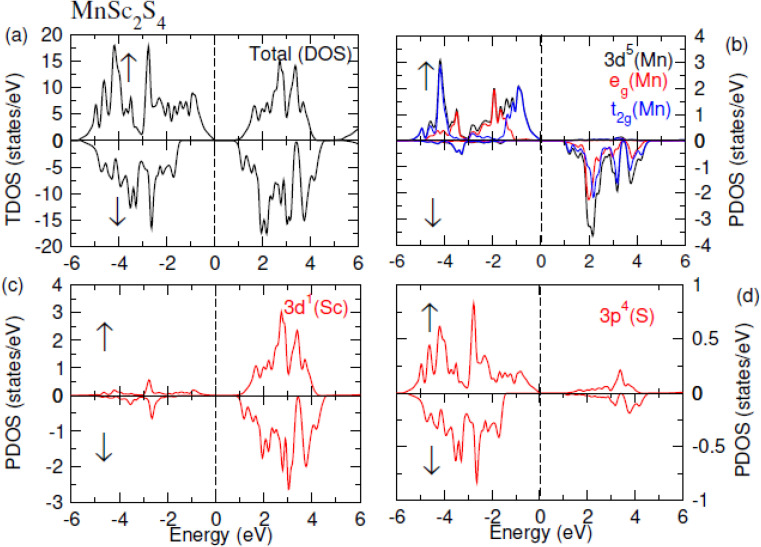
(a) TDOS plot of MnSc2S4, PDOS plots of (b) Mn, (c) Sc and (d) S.

**Fig. 6:**
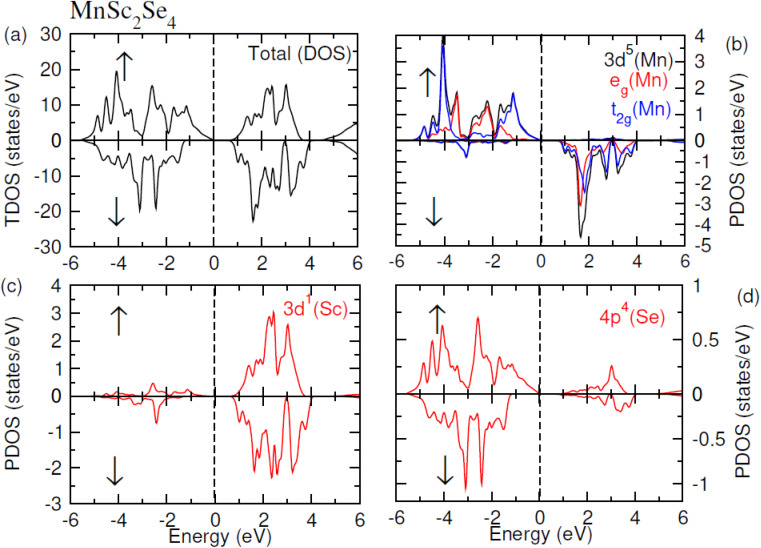
(a) TDOS plot of MnSc2Se4, PDOS plots of (b) Mn, (c) Sc and (d) Se.

The contribution of each ion in the formation of electronic bands was analyzed by plotting the TDOS and PDOS, which correspondingly represent the total and partial density of states. The Fig. 5(a) and 6(a) show the TDOS while Fig. 5(b–d) and 6(b–d) represent the PDOS plots of MnSc_2_S_4_ and MnSc_2_Se_4_ respectively. Both figures depict the semiconducting nature in both investigated compositions because the *E*_F_ lies between the energy gap. From these figures, it is apparent that in the up-spin configuration, VB of both compositions near the *E*_F_ is mainly composed of the 3d–t_2g_ states of Mn. Meanwhile, the contributions from the 3d-states of Sc and 3p/4p-states of S/Se are comparatively small. In the same spin configuration, the CB minima are mainly shaped by the major contribution of the 3d-states of Sc. There is a minor and negligible contribution of the 3p/4p-states of S/Se and 3d-states of Mn, respectively. In the spin-down configuration, the main portion of the VB maxima is formed by the participation of the 3p/4p-states of S/Se. Aside from this, near *E*_F_, a minor contribution can be seen by the 3d-states of Sc and Mn. Most of the CB minima is formed from contributions of the 3d–t_2g_ and 3d–e_g_ states of Mn and the 3d-states of Sc. However, very little is contributed by the 3p/4p states of S/Se.

### 3.4 Optical properties

**Fig. 7:**
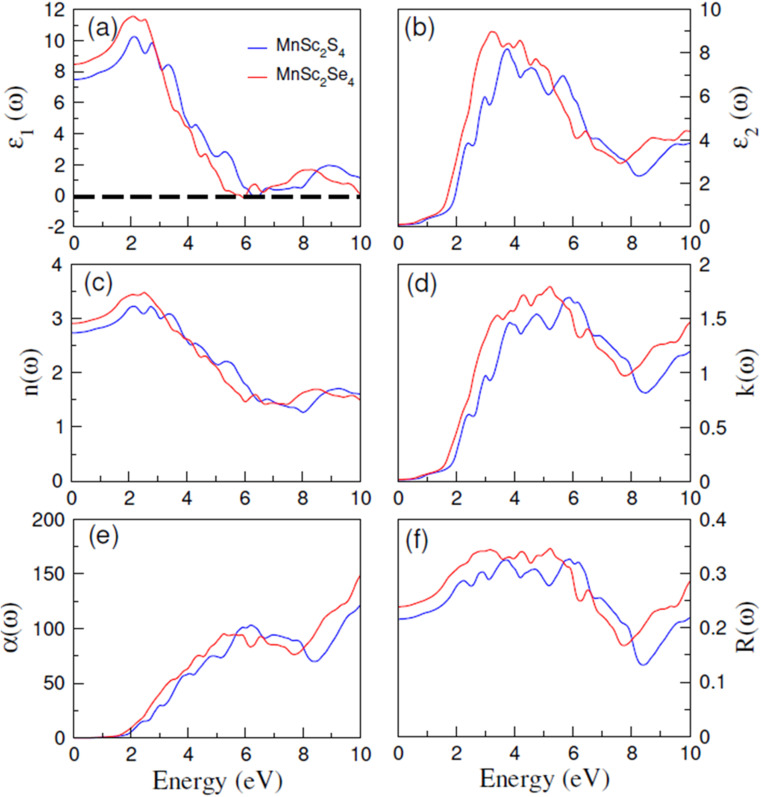
Calculated values of (a) *ε*_1_(*ω*), (b) *ε*_2_(*ω*), (c) *n*(*ω*), (d) *k*(*ω*), (e) *α*(*ω*), and (f) *R*(*ω*) for spinels MnSc_2_X_4_ (X = S, Se) in the energy range of 0 to 10 eV.

For semiconductor materials, it is critical to understand their optical properties because of their role in the overall optoelectronic performance of such materials. In pursuit of this, the present study reports on the various optical parameters, which include the dielectric constant (*ε*), refractive index (*ñ*), absorption coefficient (*α*), and reflectivity (*R*), computed for both investigated materials. All the stated parameters were frequency-dependent and computed for the energy ranges from 0 to 10 eV. The first computed parameter is *ε*, which is associated with the decrease of the electromagnetic (EM) radiation in the material. It is a complex quantity and can be written as *ε*(*ω*) = *ε*_1_(*ω*) + *iε*_2_(*ω*). The real part *ε*_1_(*ω*) corresponds to the strength of the polarization in the materials, and the imaginary part *ε*_2_(*ω*) is associated with dielectric losses. Fig. 7(a) illustrates the computed plots of *ε*_1_(*ω*), and revealed the value of static dielectric constant *ε*_1_(0) of 6.5 and 8.5 for MnSc_2_S_4_ and MnSc_2_Se_4_, respectively. The dielectric constant is related inversely with the bandgap such that *ε*_1_(0) ∝ 1/*E*_g_^2^.^34,35^ Thus, there is a relatively greater magnitude of *ε*_1_(0) in the Se-based composition. Furthermore, at optical frequencies, *ε*_1_(*ω*) decreases, which is due to the absence of dipolar polarization at those frequencies. In addition, *ε*_1_(*ω*) has a positive magnitude for both compositions throughout the frequency range. *ε*_1_(*ω*) is related to the imaginary part *ε*_2_(*ω*) through the Krammer–Koning's relations:^36^8
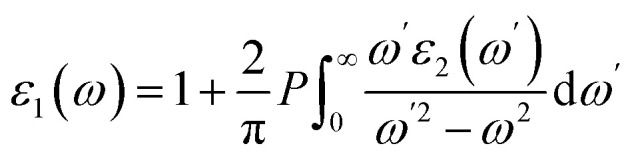


*ε*_2_(*ω*) can be calculated by utilizing the following relation:9
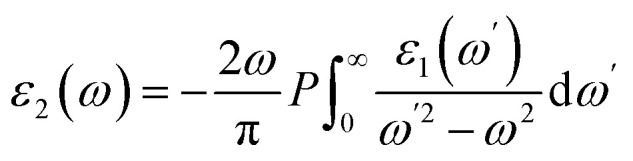
Here, *P* is the Cauchy principal value. Fig. 7(b) presents the plots of *ε*_2_(*ω*), which revealed that once the energy of the EM radiation exceeds the *E*_g_ values of both spin-configurations, the *ε*_2_(*ω*) gives the steeper peaks. This can be explained in terms of losses due to the strong absorption at energies greater than *E*_g_. The maximum magnitude of *ε*_2_(*ω*) is obtained at 3.3 and 3.7 eV for MnSc_2_S_4_ and MnSc_2_Se_4_, respectively.

There is another complex optical parameter named the refractive index (*ñ*), which can be expressed as: *ñ*(*ω*) = *n*(*ω*) + *ik*(*ω*). The real part *n*(*ω*) equals the ratio of the speed of EM radiation in space to its speed in the medium. The imaginary part *k*(*ω*), also named the extinction coefficient, quantizes the loss of EM radiation per unit distance in the medium caused by absorption and scattering.^37^Fig. 7(c) and (d) illustrate the computed plots of *n*(*ω*) and *k*(*ω*), respectively. The magnitude of the real part of the refractive index for static field *n*(0), as illustrated by the plots, is 2.75 and 2.9 for MnSc_2_S_4_ and MnSc_2_Se_4_, respectively. Its magnitude increases up to 2.5 eV, and then begins to decrease. Conversely, the plots of *k*(*ω*) give the peaks at the energy of 5.9 eV and 5.0 eV in the case of the S and Se-based compositions, respectively. The quantities *n*(*ω*) and *k*(*ω*) are linked with the dielectric constant by the relations: *ε*_1_ = *n*^2^ + *k*^2^ and *ε*_2_ = 2*nk*, respectively. Thus, *n*(*ω*) follows the same trend as that of *ε*_1_(*ω*), and *k*(*ω*) shows a trend that is almost similar to *ε*_2_(*ω*).

For semiconductors, the amount of light absorbed per unit distance in the material is very crucial to compute because the inter-band transition is associated with the absorption of light. To estimate this absorption, we compute the absorption coefficient (*α*), and its plots are illustrated in Fig. 7(e). The material only absorbs those photons of light having energy at least equal to the bandgap. It is apparent from the plots that once the energy of the EM radiation exceeds the bandgap value of both spin configurations, the absorption becomes stronger. Furthermore, the plots reveal that the two compositions exhibit strong absorption in the visible and ultraviolet (UV) regions of the spectra with most of the absorption in the UV region. For optoelectronic materials, the loss of EM radiation due to reflectance is important to note because it affects the device's performance. The reflectivity *R*(*ω*) for both materials was computed and is plotted in Fig. 7(f). The magnitude of *R*(0) for MnSc_2_S_4_ and MnSc_2_Se_4_ is 0.22 and 0.24, which increases with the energy of the EM radiation, and has a maximum magnitude of 0.345 and 0.33, respectively, in the visible and near-UV regions.

### 3.5 Magnetic properties

To analyze the magnetic behaviors observed in MnSc_2_S_4_ and MnSc_2_Se_4_, several key parameters have been thoroughly investigated, which include the exchange interactions, crystal field effects, and associated parameters. The observed variations in the bandgaps between the spin channels signify exchange splitting, resulting from the interactions between Mn and the anions (S or Se). The introduction of the S/Se atoms generates a crystal field, which splits the Mn 3d orbitals into five distinct energy levels. Among these, the nonlinear doublet e_g_, comprising (d_*z*^2^_, d_*x*^2^−*y*^2^_), experiences a greater energy increase compared to the linear triplet t_2g_, which includes (d_*xy*_, d_*yz*_, d_*zx*_). The bonding e_g_ states are energetically lower than the antibonding t_2g_ states, with the energy gap between them termed as the crystal field splitting energy (*Δ*_CF_). Within the triplet states, the d_*x*^2^−*y*^2^_ orbital has higher energy compared to the d_*yz*_ and d_*zx*_ orbitals, influencing the degeneracy patterns. This configuration aligns with Hund's rule, where unpaired electrons participate in hybridization. Such hybridization involves interactions between the Mn 3d orbitals and S/Se p orbitals, supporting the observed ferromagnetic behavior.

The energy band distribution shows that as a consequence of the exchange mechanism, the t_2g_ states migrate to reduced energies and the e_g_ states move to higher energies. Consequently, the lower-energy bonding states, which accommodate six 3d electrons of Mn, become occupied. A key parameter, the indirect exchange-splitting *Δ*_*x*_(pd), is calculated by the location of the p-states of the anion within the down-spin configuration. Its value can be calculated using the Density of States (DOS) or band structure analysis, following the relation *Δ*(pd) = *E*^↓^_V_ − *E*^↑^_V_. A negative value for *Δ*(pd) signifies that the spin-down channel experiences stronger attraction compared to the spin-up channel. This behavior is a hallmark of spin-polarized materials, reflecting the asymmetry in energy levels due to spin-dependent interactions (Table 2).

**Table 2:** Magnitude of anisotropy (*A*) in the computed elastic moduli (*Y* and *G*) and Poisson's ratios for MnSc_2_X_4_ (X = S, Se)

Parameters	Linear compressibility (*β*) (TPa^−1^)			Young's modulus (*Y*) (GPa)			Shear modulus (*G*) (GPa)			Poisson's ratio (*ν*)		
	*β*_min_	*β*_max_	*A*	*Y*_min_	*Y*_max_	*A*	*G*_min_	*G*_max_	*A*	*ν*_min_	*ν*_max_	*A*
MnSc_2_S_4_	244 230	244 230	1	0.0070567	0.01122	1.59	0.0055279	0.043178	7.811	−0.91057	−0.36172	0.3973
MnSc_2_Se_4_	197 150	197 150	1	0.007373	0.014479	1.964	0.0047678	0.099602	20.89	−0.98827	−0.22679	0.2295

The analysis sheds light on the magnetic properties of MnSc_2_S_4_ and MnSc_2_Se_4_ by incorporating the crystal field and exchange splitting, and the redistribution of electronic states, characterized by the *Δ*_CF_ for the up-spin and down-spin channels. Table 3 outlines the exchange splitting energy (*Δ*_*x*_d), derived from the peak positions of the d-states in the down-spin (↓) and up-spin (↑) channels. The comparison between *Δ*_CF_ and *Δ*_*x*_d reveals that *Δ*_*x*_d is substantially greater compared to *Δ*_CF_, highlighting the dominance of the ferromagnetic interactions over magnetic frustration in these compounds. This interplay between the exchange energies and crystal field effects underscores the mechanisms driving their magnetic behavior (Table 4).

**Table 3:** Estimated magnitudes of *Δ*_CF_, *Δ*_*x*_(*d*), *Δ*_*x*_(pd), p–d and s–d exchange constants *N*_∘_*β* and *N*_∘_*α*, respectively, for MnSc_2_S_4_ and MnSc_2_Se_4_

Parameters	MnSc_2_S_4_	MnSc_2_Se_4_
*Δ*_CF_	3.8	3.2
*Δ*_*x*_(d)	6.1	5.6
*Δ*_*x*_(pd)	−1.5	−1.2
*N*_∘_ *α*	0.12	0.07
*N*_∘_ *β*	−0.72	−0.58

**Table 4:** Up-spin bandgap ↑*E*_g_(eV), net, and the local magnetic moments (in Bohr magneton) computed for MnSc_2_X_4_ (X = S and Se)

	↑*E*_g_ (eV)	Total (μ_B_)	Int. (μ_B_)	Mn (μ_B_)	Sc (μ_B_)	X (μ_B_)
MnSc_2_S_4_	0.72	5.0000	0.9700	4.1708	0.1209	0.0256
MnSc_2_Se_4_	0.56	5.0000	1.0041	4.1566	0.1254	0.0211

The exchange interaction between the p orbitals of S/Se and the 3d orbitals of Mn generates exchange energy due to p–d coupling. The stabilization of ferromagnetism arises from the negative magnitude of the exchange energies, which reduce the overall energy of the system. The coupling of the conduction electrons and valence band holes with the d-orbitals of Mn is characterized by the s–d and p–d exchange constants depicted by *N*_∘_*α* and *N*_∘_*β*, respectively. The magnitude of these constants can be determined by the following expressions:10
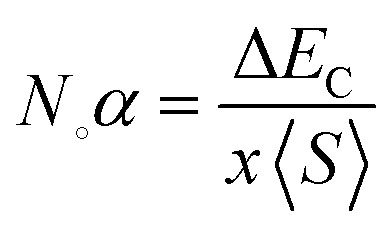
11
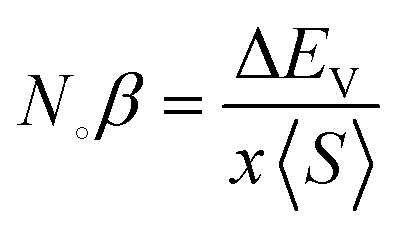
Here, (*x*) represents the degree of Mn participation, and 〈*S*〉 denotes the average magnetic moment. The negative magnitude of the indirect exchange energy at the edge of the valence band in the down-spin configuration reflects the system's energy minimization by means of double–exchange interaction. To further explore the ferromagnetic (FM) behavior, the splitting at the edges of the conduction band (Δ*E*_C_ = *E*^↓^_C_ − *E*^↑^_C_) and the edges of the valence band (Δ*E*_V_ = *E*^↓^_V_ − *E*^↑^_V_) is analyzed. These energy differences provide insights into the FM mechanism, with Table 3 summarizing the calculated exchange constants, which include both positive and negative values. In ferromagnetic systems, the negative exchange energy in the down-spin configuration plays a dominant role, consistent with the characteristics of indirect exchange interactions.

It is determined from the spin-dependent volume optimization that the investigated materials are FM in nature. However, the strength of the magnetization of the materials was evaluated by the measurement of their magnetic moment. The magnetic moment emerges due to the spin-polarized electrons, and it can be computed by the difference in the integral of spin-polarized DOS. In addition to the net magnetic moment, the local magnetic moment provided by individual ions in both compositions was computed and are listed in Table 3. Both compositions exhibit identical net magnetic moments with the magnitude of 5.000 μB, in which Mn alone contributes the magnetic moment of 4.1705 and 4.1566 μB in MnSc_2_S_4_ and MnSc_2_Se_4_, respectively. The reason for the high contribution of Mn is the unpaired electrons in its d-orbitals. This can also be visualized by the spin-polarized PDOS of the Mn ion, which shows the majority of the occupied up-spin states in the valence band. Aside from Mn, the contribution of Sc and S/Se is comparatively negligible. However, a decent portion of the magnetic moment comes from the interstitial region, which was also computed and tabulated in Table 3.

### 3.6 Thermoelectric properties

The major challenge with energy harvesting systems is their limited efficiency due to energy losses in the form of heat. In this regard, thermoelectric (TE) materials, capable of harvesting electrical energy from waste heat, are crucial to increase the efficiency of existing energy generation systems. In addition, these materials can be used as independent energy harvesting systems for sustainable energy solutions. Considering this, in the reported investigation, we computed the transport characteristics of MnSc_2_S_4_ and MnSc_2_Se_4_ using the BoltzTrap code. The reported transport parameters include the electrical and thermal conductivities represented by *σ* and *κ*_e_, respectively, and the Seebeck coefficient (*S*). All the mentioned parameters were computed against varying temperatures from 300 to 800 K. Since the spin-polarized nature of the materials leads to different magnitudes of the computed parameters in different spin orientation, the following equations were used to compute the net magnitude of these parameters.^38^12
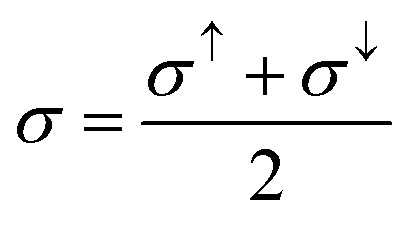
13
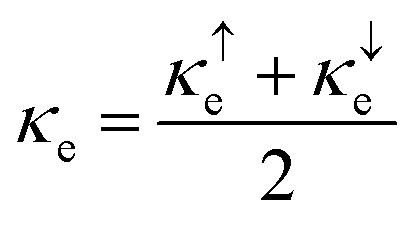
14
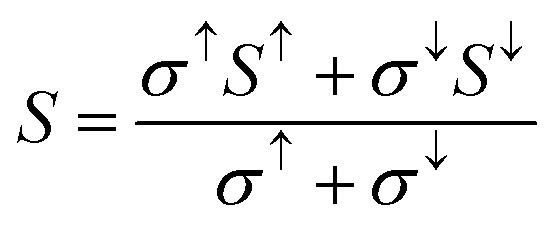


**Fig. 8:**
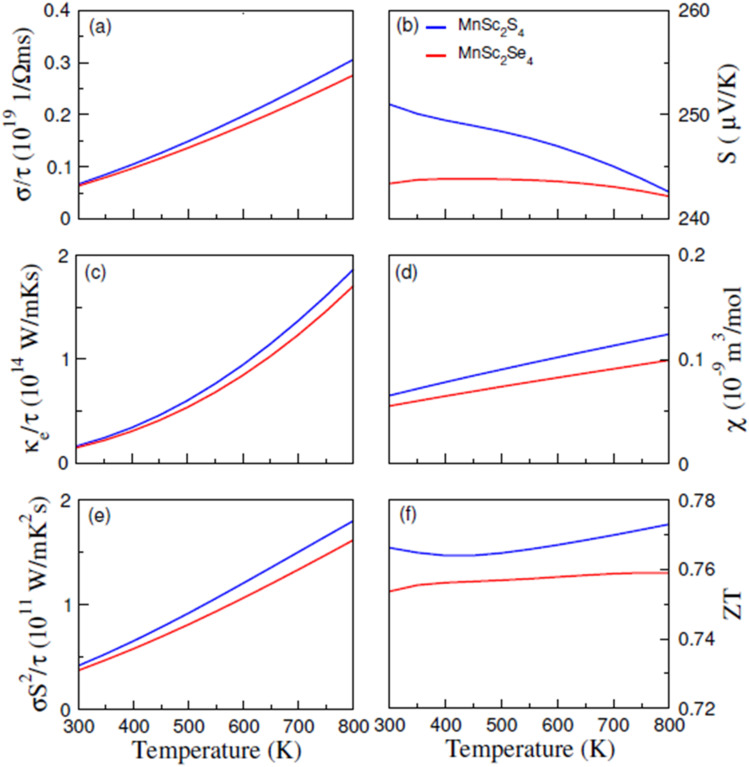
Estimated plots of (a) *σ*/*τ*, (b) *S*, (c) *k*_e_/*τ*, (d) *χ*, (e) P.F. and (f) *ZT* against increasing temperatures for spinel compounds MnSc_2_X_4_ (X = S, Se).

**Fig. 9:**
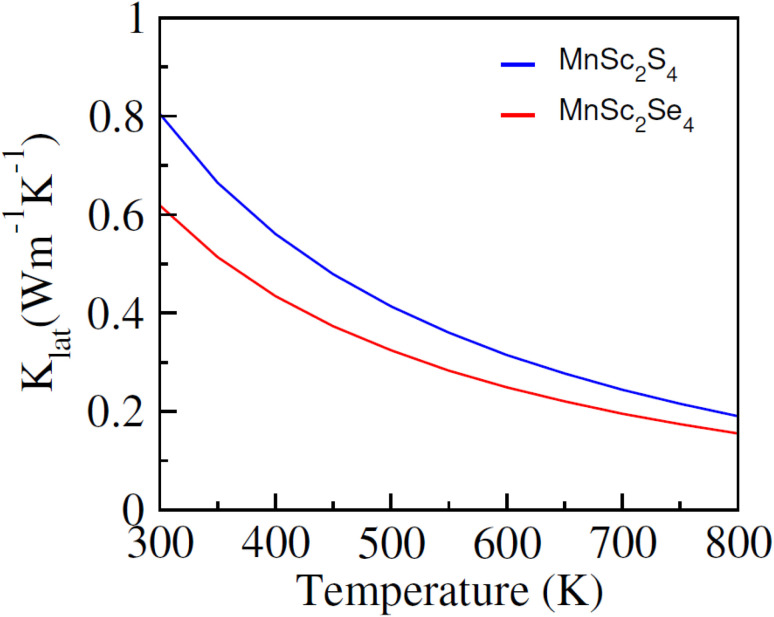
Computed lattice conductivity (*k*_lat_) for spinel compounds MnSc_2_X_4_ (X = S, Se) against temperature.

Fig. 8(a) illustrates the computed plots of *σ*/*τ*, which revealed that its magnitude increases almost linearly with temperature. The semiconducting nature of the composition accounts for this increasing trend. The increasing temperature supplies thermal energy to the materials, enough to excite the electrons to the conduction band and hence increase the carrier concentration, which in turn increases the electrical conductivity. At 300 K, the computed magnitude of *σ*/*τ* is 0.066 × 10^19^ (Ω ms)^−1^ and 0.07 × 10^19^ (Ω ms)^−1^, which increases and reaches its maximum at 800 K with the magnitude of 0.307 × 10^19^ (Ω ms)^−1^ and 0.278 × 10^19^ (Ω ms)^−1^ for MnSc_2_S_4_ and MnSc_2_Se_4_, respectively. In regards to thermal conductivity, it comprises two components: *κ* = *κ*_e_ + *κ*_L_; where, *κ*_e_ represents the part that is contributed by the charge carriers, and *κ*_L_ signifies the part that comes from the lattice vibrations. The *κ*_e_/*τ* was computed using the BoltzTrap code and its plots are elucidated in Fig. 8(c), which show a roughly similar increasing trend as with *σ*/*τ*. At 300 K, the magnitude of *κ*_e_/*τ* is 0.17 × 10^14^ W mK^−1^ s^−1^ for both MnSc_2_S_4_ and MnSc_2_Se_4_, which reaches its maximum value of 1.87 × 10^14^ and 1.7 × 10^14^ W mK^−1^ s^−1^ at 800 K, respectively. Moreover, the lattice part of the thermal conductivity (*κ*_L_) has been investigated by employing ShengBTE code,^39^ as illustrated in Fig. 9. The magnitudes of *κ*_L_ for MnSc_2_S_4_ and MnSc_2_Se_4_ gradually decrease from 0.80 and 0.61, respectively, when the temperature increases from 200 to 800 K. The magnitudes of *κ*_L_ are particularly inferior to *κ*_e_, indicating that the thermoelectric efficiency is not decisively affected by the *κ*_L_ of MnSc_2_S_4_ and MnSc_2_Se_4_. Thus, the performance analysis factor *ZT* is slightly altered by *κ*_L_.

When a TE material is exposed to a temperature gradient, then a voltage is generated across its terminals quantified by the Seebeck coefficient (*S*). The parameters that influence the magnitude of *S* are given in the following equation:^40^15
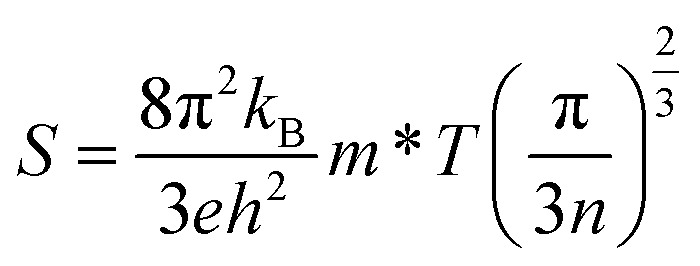
In this relation, *k*_B_, *h*, and *e* are constant quantities and represent the Boltzmann constant, Planck's constant, and charge on the charge carrier. *T*, m* and *n* denote the absolute temperature, carrier's effective mass and carrier concentration, respectively.

Fig. 8(b) depicts the plots of *S*, computed for MnSc_2_S_4_ and MnSc_2_Se_4_. Both compositions have a good value of *S*, which is greater than 240 μV K^−1^, for the whole temperature range. Furthermore, the positive values of *S* signify the p-type nature of the materials. For MnSc_2_S_4_, the graph shows the magnitude of 251 μV K^−1^ at 300 K, which decreases non-linearly and becomes 242.5 μV K^−1^ at 800 K. The increase in the carrier concentration (*n*) caused by the increasing temperature is the reason for this decrease in *S*. In the case of MnSc_2_Se_4_, the magnitude of *S* at 200 K is 243 μV K^−1^, which has a negligible net change with temperature, and becomes 242 μV K^−1^ at the temperature of 800 K. It seems that the decrease in the value of *S* due to the increasing carrier concentration is neutralized with the increase in its magnitude due to temperature.

Since, the materials showed magnetic behavior, their ability to be magnetized when exposed to an external magnetic field can be analyzed by the parameter called magnetic susceptibility (*χ*). The variation in *χ* against the increasing temperature was computed, and is illustrated in Fig. 8(d). The plots demonstrate that both compositions have nearly identical linear increases in the *χ* with temperature. At 800 K, the maximum values of the *χ* for S and Se-based composition are 0.125 × 10^−9^ and 0.1 × 10^−9^ m^3^ mol^−1^, respectively. We assess the efficiency of the materials in harvesting energy from heat using a parameter known as the power factor (P.F. = *σS*^2^/*τ*). Fig. 8(e) presents the plots of the computed P.F., which unveil the increasing trend with temperature. This increasing trend comes from the increased electrical conductivity at high temperatures. For MnSc_2_S_4_ and MnSc_2_Se_4_, the magnitude of P.F at 300 K is 0.4 × 10^11^ and 0.35 × 10^11^ W mK^−2^ s^−1^, which increases and reaches its maximum at 800 K with the value of 1.8 × 10^11^ and 1.64 × 10^11^ W mK^−2^ s^−1^, respectively. At the end, the overall TE functionality of the investigated spinels was probed through the dimensionless variable named the figure of merit (*ZT*), which is calculated by using the previously computed parameters, such that *ZT* = (*S*^2^*σ*/*κ*_e_)*T*. The plots in Fig. 8(f) depict a high *ZT* value of 0.76 to 0.77 with a negligible variation throughout the temperature range, making the material equally effective for room and high-temperature TE applications.

### 3.7 Phonon dispersion plot

**Fig. 10:**
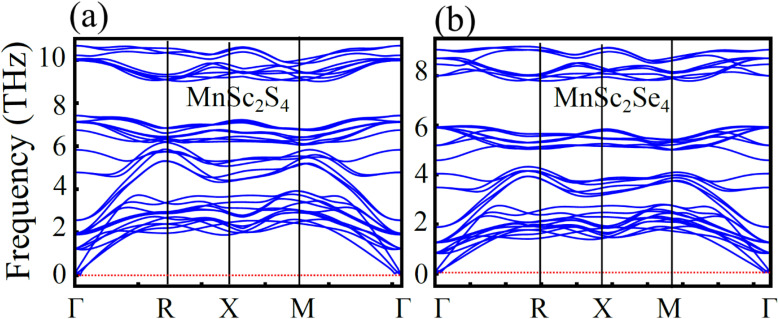
Phonon dispersion for spinel compounds (a) MnSc_2_S_4_ and (b) MnSc_2_Se_4_.

To probe the dynamic stability, calculations of the phonon dispersion along the high-symmetry point within the Brillouin zone were performed, and the phonon dispersion curves are plotted in Fig. 10. The plot demonstrates that all of the vibrational modes are positive in the Brillouin zone for MnSc_2_S_4_ and MnSc_2_Se_4_, which is an indication of the compounds' dynamic stability. On the other hand, the existence of negative frequencies is indicator of instability in some forms of the vibrational states, but no such frequency is depicted in the computed plots.

## 4. Conclusion

A detailed investigation of the mechanical, optoelectronic, and magnetic properties of the chalcogenide spinels MnSc_2_X_4_ (X = S, Se) was conducted by employing the density functional theory. The spinels were observed to have cubic structures with the *Fd*3̄*m* space group. The mechanical stability of the spinels was validated by the calculated magnitudes of elastic stiffness constants. Furthermore, their ductile nature was attested by the computed Poisson's and Pugh's ratios, which exhibited magnitudes greater than 0.26 and 1.75, respectively. The thermodynamic stability was assessed by the formation energies (Δ*H*_f_), which were −1.45 and −1.34 eV in the case of MnSc_2_S_4_ and MnSc_2_Se_4_ respectively. The negative magnitude revealed that formation involves an exothermic reaction, which confirmed the thermodynamic stability. Furthermore, the S-based composition had a greater magnitude of Δ*H*_f_, which made it relatively more stable. The spin-polarized EBS and DOS demonstrated the semiconducting behavior with the direct bandgap of 0.72 and 0.56 eV in the spin-up orientation for MnSc_2_S_4_ and MnSc_2_S_4_, respectively. Furthermore, the materials exhibited high absorption in the visible to UV region of the spectrum, making them suitable for solar cells and UV-detection applications. In terms of their magnetic characteristics, the materials exhibit a ferromagnetic nature with a magnetic moment of 5.000 μB, which was primarily attributed to the Mn ions. The thermoelectric characteristics were also analyzed by BoltzTrap code, which revealed the high Seebeck coefficient in the range of 242 to 251 μV K^−1^ for the temperature range of 300 to 800 K. With increasing electrical conductivity and a decent figure of merit, the materials show potential for thermoelectric applications. Overall, the outcomes of the study demonstrate that the materials are suitable for optoelectronic and thermoelectric applications.

## Data availability

All data included in this research will be made available upon request.

## Conflicts of interest

There is no conflict to declare.
